# Efficacy of ChatGPT in Cantonese Sentiment Analysis: Comparative Study

**DOI:** 10.2196/51069

**Published:** 2024-01-30

**Authors:** Ziru Fu, Yu Cheng Hsu, Christian S Chan, Chaak Ming Lau, Joyce Liu, Paul Siu Fai Yip

**Affiliations:** 1 The Hong Kong Jockey Club Centre for Suicide Research and Prevention Faculty of Social Sciences The University of Hong Kong Hong Kong SAR China (Hong Kong); 2 Department of Psychology The University of Hong Kong Hong Kong SAR China (Hong Kong); 3 Department of Psychology and Linguistics International Christian University Tokyo Japan; 4 Department of Linguistics and Modern Language Studies The Education University of Hong Kong Hong Kong SAR China (Hong Kong); 5 Department of Social Work and Social Administration Faculty of Social Sciences The University of Hong Kong Hong Kong SAR China (Hong Kong)

**Keywords:** Cantonese, ChatGPT, counseling, natural language processing, NLP, sentiment analysis

## Abstract

**Background:**

Sentiment analysis is a significant yet difficult task in natural language processing. The linguistic peculiarities of Cantonese, including its high similarity with Standard Chinese, its grammatical and lexical uniqueness, and its colloquialism and multilingualism, make it different from other languages and pose additional challenges to sentiment analysis. Recent advances in models such as ChatGPT offer potential viable solutions.

**Objective:**

This study investigated the efficacy of GPT-3.5 and GPT-4 in Cantonese sentiment analysis in the context of web-based counseling and compared their performance with other mainstream methods, including lexicon-based methods and machine learning approaches.

**Methods:**

We analyzed transcripts from a web-based, text-based counseling service in Hong Kong, including a total of 131 individual counseling sessions and 6169 messages between counselors and help-seekers. First, a codebook was developed for human annotation. A simple prompt (“Is the sentiment of this Cantonese text positive, neutral, or negative? Respond with the sentiment label only.”) was then given to GPT-3.5 and GPT-4 to label each message’s sentiment. GPT-3.5 and GPT-4’s performance was compared with a lexicon-based method and 3 state-of-the-art models, including linear regression, support vector machines, and long short-term memory neural networks.

**Results:**

Our findings revealed ChatGPT’s remarkable accuracy in sentiment classification, with GPT-3.5 and GPT-4, respectively, achieving 92.1% (5682/6169) and 95.3% (5880/6169) accuracy in identifying positive, neutral, and negative sentiment, thereby outperforming the traditional lexicon-based method, which had an accuracy of 37.2% (2295/6169), and the 3 machine learning models, which had accuracies ranging from 66% (4072/6169) to 70.9% (4374/6169).

**Conclusions:**

Among many text analysis techniques, ChatGPT demonstrates superior accuracy and emerges as a promising tool for Cantonese sentiment analysis. This study also highlights ChatGPT’s applicability in real-world scenarios, such as monitoring the quality of text-based counseling services and detecting message-level sentiments in vivo. The insights derived from this study pave the way for further exploration into the capabilities of ChatGPT in the context of underresourced languages and specialized domains like psychotherapy and natural language processing.

## Introduction

### Overview

With the rise in mental health demands and challenges, technology-enabled text-based counseling services and therapy are increasingly popular, especially among younger generations [1]. Compared with traditional counseling, web-based services afford higher degrees of accessibility and anonymity [2,3]. In addition to these advantages, web-based, text-based counseling can potentially provide complete electronic records for downstream analyses.

Developing tools to systematically analyze text data is consequently important to enable quantitative evaluation of the service and assist practitioners in making clinical decisions. As a subfield of artificial intelligence (AI) and linguistics, natural language processing (NLP) enables computational approaches to learn, interpret, process, and analyze human language in written or spoken form, making it a promising tool to analyze counseling text [4]. Althoff et al [5] used text features to understand and evaluate counselor performance and counseling outcomes. Broadbent et al [6] developed NLP techniques to identify suicide risks in text-based crisis counseling records. Xu et al [7] constructed a word affinity network to predict suicidal ideation before its emergence in the context of a web-based, text-based counseling service. These studies not only leveraged the nature of electronic counseling records to conduct clinical research but also applied NLP techniques to understand psychotherapy at a scale previously unattainable.

Sentiment analysis is one of the most active research areas in NLP, leading to its rapid development with the help of machine learning models [8]. Sentiment analysis focuses on identifying the sentiments, emotions, or opinions expressed in texts, such as social media posts, conversations, or user reviews. In counseling, sentiment is an important variable. Althoff et al [5] used anonymized counseling sessions from a crisis intervention service to study how various linguistic aspects correlate with session outcomes. After each session, the help-seeker received a follow-up question: “How are you feeling now? Better, same or worse” which was used as the session quality indicator. Linguistic Inquiry and Word Count (LIWC) was used to measure help-seeker sentiments throughout counseling sessions, and the authors found that constructive changes in perspective throughout the session were related to a higher session quality. Furthermore, sentiment analysis has the potential to objectively and automatically evaluate counselors’ expressed empathy, which is key in counseling and mental health support [9]. With its potential use in counseling contexts, there is a pressing need to develop reliable NLP tools for sentiment analysis applicable to such domains.

In recent years, large language models (LLMs), which are pretrained on extremely large amounts of text data and encompass over a hundred billion parameters, have profoundly affected research communities with their impressive general NLP ability in various tasks, including sentiment analysis. ChatGPT stands out as a powerful LLM for accomplishing question-answering tasks. Several studies have explored ChatGPT’s capability of understanding sentiments in different languages. For example, Rathje et al [10] used GPT-3.5 and GPT-4 to conduct 3 sentiment analysis tasks (ie, sentiment, discrete emotion, and offensiveness) across 12 languages. They found that GPT was effective in multilingual text analysis. Wang et al [8] also demonstrated that ChatGPT is effective in sentiment analysis through 5 sentiment analytic tasks in English. Bang et al [11] tested ChatGPT’s strengths and limitations in reasoning and interactivity across 4 languages. They concluded that ChatGPT outperformed other LLMs in most tasks but suffered hallucinations (ie, producing untruthful content that contradicts existing sources or lacks supporting evidence [12]). To date, few studies have evaluated ChatGPT’s capability for analyzing Cantonese texts.

This study seeks to validate ChatGPT as a tool for sentiment analysis in Cantonese. Cantonese is a Chinese language under the Yue branch, with over 73 million speakers worldwide [13]. Its distinctive linguistic features pose unique challenges to NLP, particularly to sentiment analysis.

The first of these linguistic features of Cantonese is that it shares the Chinese ideographic writing system. The apparent similarity between Cantonese and Standard Chinese can lead to high inaccuracy in Cantonese sentiment analysis tasks. For example, the same words in Cantonese and Standard Chinese can have drastically different meanings, for example, 得意 (in Cantonese: cute, and in Standard Chinese: proud).

Second, despite the high similarity between Cantonese and Standard Chinese, Cantonese can differ significantly in grammatical and lexical features from Standard Chinese. For example, grammatical elements that indicate temporal references, certainty, questions, agreements, and so on, can be different between Cantonese and Standard Chinese and can thus lead to misinterpretation. Moreover, there are thousands of characters unique to Cantonese [14]. The sentiments carried by these characters could potentially be neglected. For example, in Cantonese, the specific words that express negation (唔 m4, 冇 mou5) are not found in Standard Chinese, for which LLMs may incorrectly determine a clause as affirmative or negative.

The third linguistic peculiarity is that, compared with Standard Chinese, which is mainly used in formal writing, Cantonese is mostly informal and colloquial. In regions such as Hong Kong, Cantonese, when used in online communication channels, including social media platforms, forums, and online chats, is more informal and colloquial compared with its use in formal writing or speech, which often contains more typos or grammatical peculiarities [15]. The informal and colloquial nature of Cantonese poses significant challenges for NLP to capture the intended sentiments. Moreover, Cantonese has historically evolved in a multilingual environment, especially Hong Kong Cantonese, which contains many borrowed words from English through phonetic transliteration [13]. Therefore, the challenges in Cantonese sentiment analysis are varied and deeply rooted in the language’s linguistic peculiarities. Cantonese is widely used yet distinct from other languages; it is thus a valuable case study.

Many effective and advanced NLP techniques focus on high-resource languages, such as English and Mandarin Chinese. The NLP techniques for these languages are much more developed than those for other languages because of their popularity and the availability of labeled data and expert resources. Under the taxonomy of Joshi et al [16], Cantonese is considered a Tier-1 language, which indicates that it is a low-resource language in the field of NLP. This categorization reflects the lack of labeled data sets and specific NLP resources despite its strong web presence and quantity of users, which suggests the availability of a wealth of unlabeled data. As a result, to date, limited studies have been conducted on Cantonese NLP tasks [17].

To help fill this gap, in this study, ChatGPT’s performance on Cantonese sentiment analysis tasks was evaluated and compared with other state-of-the-art models. We asked ChatGPT to perform sentiment analysis on the digital transcripts from a web-based, text-based counseling service in Hong Kong.

### Background and Related Works

#### Cantonese Sentiment Analysis

Sentiment analysis can be categorized into 2 approaches: the lexicon-based approach of building a large corpus of different sentiments and the machine learning approach mixed with the lexicon-based approach. Both methods have their advantages and disadvantages in terms of preparing data for training, inferencing time, and accuracy. The lexicon approach is less used in Cantonese sentiment analysis tasks. So far, only Chen et al [18] used the hidden Markov model to perform word segmentation and then built a sentiment word dictionary for Cantonese. No study was conducted to establish the effectiveness of this model.

In recent years, machine learning approaches, mixed with lexicon-based knowledge, have shown promising results in sentiment detection tasks. Three studies have used supervised learning mixed with lexicon-based methods to classify sentiments in Cantonese text. Ngai et al [17] used a combination of 3 popular classifiers: support vector machine (SVM) with naive Bayes feature-based, convolutional neural network-based, and lexicon-based classifiers. The authors first manually built a sentiment word dictionary using many sources, such as the “Yue Dictionary” [19]. They then trained the combination of the 3 classifiers with sentiment features on massive Cantonese text data from social media sites, including Facebook and Weibo. Subsequently, the model was tested on users’ reviews on Weibo, Facebook, and Dianping. The *F*_1_-score of the approach was approximately 72%. Xiang et al [20] explored the deep learning model of long short-term memory (LSTM) combined with an attention mechanism, namely “long short-term memory sentiment augmented attention network” (LSTM-SAT), to classify reviews on OpenRice, a Hong Kong restaurant search website that allows users to post their ratings and reviews. The accuracy of their model was 60.8%. Before modeling, they used an automatic method to expand a manually crafted Cantonese sentiment word dictionary. In addition, pretrained language models (PLMs), which are first pretrained on an extensive corpus using unsupervised learning and then fine-tuned in downstream tasks with a relatively small data set using supervised learning, serve as another way to conduct Cantonese sentiment analysis. Although there is a lack of Cantonese PLMs, Tan et al [21] used the Bidirectional Encoder Representations from Transformers pretrained on a large corpus of simplified Chinese and several simplified Chinese emotional corpora to conduct Cantonese sentiment analysis, in which Cantonese text from an online social forum in Hong Kong was first translated to simplified Chinese. The reported optimal loss value of the test set was 0.065, and the optimal value of the area under the receiver operating characteristic curve was 0.996.

Among the 3 studies applying machine learning mixed with lexicon-based approaches, the first 2 both entailed building a Cantonese sentiment word dictionary and applying existing state-of-the-art machine learning models, while the third study used the latest development of PLMs from simplified Chinese, which is a high-resource language, on Cantonese, a low-resource language [16]. All 3 approaches share some disadvantages in accurately detecting sentiments. The first 2 rely on the construction of the Cantonese sentiment word dictionary, while the third approach is predicated on the quality of translation and the sentiment dictionary. The mixed-based approach yielded higher accuracy by providing extra data preprocessing (eg, Cantonese-simplified Chinese translation) and training-and-testing mechanisms to calibrate the dictionary with the context.

#### Large Language Models for Sentiment Analysis

**Figure 1:**
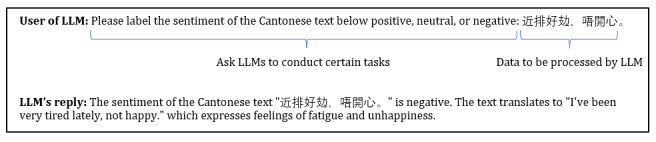
An example of a prompt and a response in a large language model (LLM).

ChatGPT is an LLM that is pretrained on extensive amounts of text data. Encompassed with over a hundred billion parameters, LLMs are capable of understanding natural human languages and generating relevant human-like responses. When using LLMs, users’ prompts often follow a 2-phase process; they first ask LLMs to conduct certain tasks, and then they provide the data to be evaluated by LLMs (Figure 1).

The LLM approach could alleviate the disadvantages in the existing literature of Cantonese sentiment analysis in subsequent ways. First, during the data preprocessing stage, there is no need to build a Cantonese sentiment word dictionary or translate Cantonese to other high-resource languages. This not only improves efficiency but also avoids potential loss in translation. Second, the LLM requires no training data, eliminating the labor-intensive training phases presented in the existing approaches. Bang et al [11] explored ChatGPT’s sentiment analysis ability across 4 languages: English, Indonesian, Javanese, and Buginese. The results show that ChatGPT outperformed the existing state-of-the-art models by a large margin for 3 of the 4 languages (ie, except Buginese). This suggests that ChatGPT possesses a certain degree of understanding of low-resource languages.

To the best of our knowledge, there is no comprehensive evaluation of ChatGPT’s ability to conduct sentiment analysis tasks in Cantonese text. In this study, we used a zero-shot prompt asking ChatGPT to perform sentiment analysis on the counseling transcript data from a Hong Kong web-based, text-based counseling service. In our context, zero-shot learning refers to the fact that no additional annotated samples were given to ChatGPT in the prompts; that is, ChatGPT performed the task solely based on our instructions. The findings could provide support for using ChatGPT to facilitate automated Cantonese sentiment analysis, which can have tremendous implications, such as harnessing ChatGPT as a cost-effective labeling and coding tool for both research and applied purposes, monitoring message-level sentiment of both service users and providers in real-time, paving the way for further studies on ChatGPT’s ability in other NLP or psychotherapy-related tasks, and transferring our presented methodologies to studies of other low-resource language contexts. Subsequently, the advantages of cutting-edge NLP technology could become accessible to the billions who communicate in those low-resource languages, unlocking immeasurable immediate benefits for them.

## Methods

### Ethical Considerations

This study was approved by the Human Research Ethics Committee at the University of Hong Kong (EA210185). Users gave their consent to the use of their text data for research purposes by accepting the privacy policy before the commencement of service. Individual service providers (ie, counselors and volunteers) gave written consent to the use of their text data for research purposes.

### Data Source

Open Up is a free 24/7 web-based, text-based counseling service in Hong Kong that enables people aged between 11 and 35 years to anonymously chat with paid staff (staff counselors or social workers) or trained volunteers [19]. In its original planning, the service aimed to provide web-based support to youth aged 24 years or younger. However, it became clear to the service partners that there was a lack of service for young adults aged between 25 and 35 years in the community. The service age range was then broadened.

Open Up counselors possess at least a bachelor’s degree in social work or counseling. Over 90% of the staff are registered social workers in Hong Kong. The age of the staff ranges from 25 to 45 years, and the female-male ratio is approximately 2:1. On average, the staff have 8 years of work experience, including 1-2 years related to web-based counseling [22]. Most trained volunteers are undergraduate students studying psychology, counseling, or social work.

**Figure 2:**
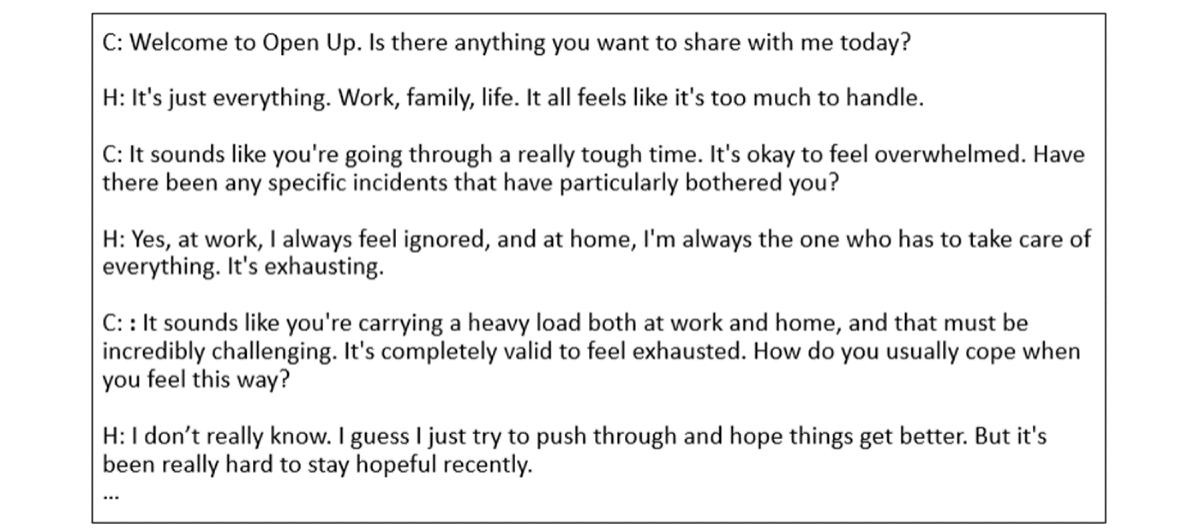
A fictitious conversation excerpt between a counselor (C) and a help-seeker (H) in Open Up.

Help-seekers can access Open Up anywhere and anytime for free through the service’s website, WhatsApp, SMS text messages, or Facebook. Due to the anonymity of the service, the help-seekers’ demographic is unknown. Since its launch in 2018, to date (May 2023), more than 224 counselors have engaged with help-seekers across more than 110,000 sessions. Figure 2 shows the opening of a fictitious session between a counselor and a help-seeker.

In this study, 342,752 counseling sessions from January 1, 2019, to May 1, 2023, were extracted, which contained 12,056,109 messages between help-seekers and counselors. In Open Up, valid sessions are defined as those with at least 4 message exchanges between the help-seeker and the counselor. This threshold was set based on a previous study [23], which found that some clients logged on to the platform without revealing any intention to use the service; these sessions are typically briefer than 4 message exchanges. Thus, 116,999 sessions with 9,390,973 messages with at least 4 message exchanges between the help-seeker and the counselor were extracted. Among them, 48,717 sessions with 3,878,653 messages in which help-seekers accessed the service for the first time were included in this study to avoid the overrepresentation of sentiments from repeated and frequent help-seekers.

We then filtered out 1342 non-Cantonese sessions that contained a total of 104,918 messages as well as 534,410 system messages (eg, “Please wait, we are connecting you to an Open Up counselor”). The exclusion of system messages was necessary because system messages could still be present in longer sessions. They were labeled as sent from counselors but were actually generated by the system. These system messages are not part of the actual counseling process. Including such messages would introduce noise to the data and potentially confound the results. Therefore, we further excluded such messages. Subsequently, 2565 sessions with fewer than four message exchanges between the help-seeker and counselor were excluded.

**Figure 3:**
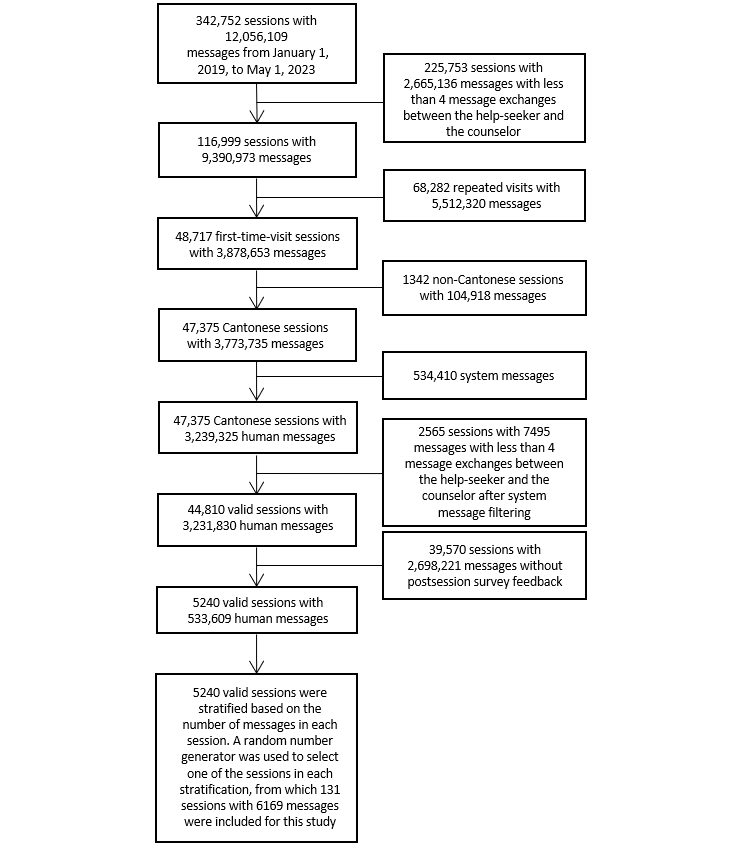
Data inclusion flow.

After filtering, there were 44,810 valid sessions containing a total of 3,231,830 messages. Only sessions with postsession survey feedback were included in this study. In total, this included 5240 sessions with 533,609 messages (Figure 3). We stratified the 5240 sessions based on the number of messages in each session. There were a total of 131 unique message count groups among the 5240 sessions. The Python package *Random* (version 3.8.2; Python Software Foundation) was used to initialize a random number generator with a fixed seed to select 1 of the sessions in each stratification, from which 131 sessions were randomly selected with 6169 messages to be included in the analyses. We decided to sample 1 session per stratum based on the available human resources for coding.

### Annotation

A codebook (Multimedia Appendix 1) developed by seasoned social workers and researchers in counseling services was used to ensure the quality of the annotations. The codebook contains rules, guidelines, examples, definitions, and tips for the annotation process. The definitions of the three sentiment categories are briefly described as follows:

1. Positive class: positive sentences describe feelings of pleasure, gratitude, satisfaction, compliment, or recommendation. Emojis and punctuation can be indicative of positivity.
2. Negative class: negative sentences describe feelings of disagreement, sadness, complaint, or hate. Factual information, such as the description of the help-seeker’s distressing issues, was also considered negative. Emojis and punctuation can also be indicative of negativity.
3. Neutral class: the neutral label is used for any sentences the annotator could not identify as positive or negative. Factual sentences that do not present sentiment orientation, such as “I am 25 years old,” and sentences the annotator cannot completely comprehend, were annotated as neutral. Sentences that are routine replies from the counselor, such as “Is there anything you want to share with me today?” were also labeled as neutral.

A total of 3 annotators labeled 500 messages from the data set to examine whether the guideline was clear. The κ agreement of the 3 annotators was 0.95, indicating a high level of agreement, and the guideline was sufficiently clear. In cases where the 3 annotators disagreed on the labeling among the 500 messages, the final label was assigned based on the majority rule among the 3 annotators. Then, one of the annotators labeled the rest of the messages, following the guidelines. Most messages with 1 or multiple sentences expressed 1 consistent sentiment. Only 35 messages out of 6169 messages (0.6%) contained a mixture of sentences expressing different sentiments. The ambiguity of the overall sentiment of such messages was discussed among the coders, and a set of rules was developed to annotate such messages’ sentiment (Multimedia Appendix 1). The count and proportion of messages categorized into the 3 sentiment classes by human annotation are as follows: positive sentiment in 15.5% (959/6169), negative sentiment in 31.5% (1943/6169), and neutral in 53% (3267/6169) messages.

### Usage of ChatGPT

Two of the most popular ChatGPT variants, GPT-3.5 (gpt-3.5-turbo) and GPT-4 (gpt-4), were used to label each message. We implemented both models with zero-shot learning. The prompt was set as “Is the sentiment of this Cantonese text positive, neutral, or negative?” The temperature of the ChatGPT models was set at 0 to generate consistent answers with the least creativity. The hyperparameter setting is shown in Table 1.

**Table 1:** Prompt settings used.

Model	GPT-3.5	GPT-4
Prompt	“Is the sentiment of this Cantonese text positive, neutral, or negative? Respond with the sentiment label only.”	“Is the sentiment of this Cantonese text positive, neutral, or negative? Respond with the sentiment label only.”
max_token	4096	8192
Engine	gpt-3.5-turbo	gpt-4
Temperature	0.0	0.0

### Baseline Details

We compared ChatGPT with a lexicon-based method and 3 machine learning models: LIWC, logistic regression (LR), SVM, and LSTM. The general procedure for training a sentiment analysis model is data preprocessing, word vector training, training and test set splitting, hyperparameter setting, model training, and evaluation.

First, according to the general framework above, we started with data pre-processing. Unlike English, there is no obvious boundary between words in Cantonese text. For example, “I feel sad” in Cantonese looks like “Ifeelsad.” Therefore, word segmentation is the first step in data preprocessing. Jieba [24], a cutting-edge word segmentation tool, was used to process each message. Next, as there are trivial words that do not carry much meaning and may introduce noise in the analyses (eg, “呢個” meaning “this” and “跟住” meaning “then”), we removed these words using a stopwords dictionary tailor-made for Open Up data. After word filtering, a corpus of 7,012,611 messages was compiled, composed of a vocabulary of 70,951 unique words, which were then used as inputs in the word vector training process.

**Figure 4:**
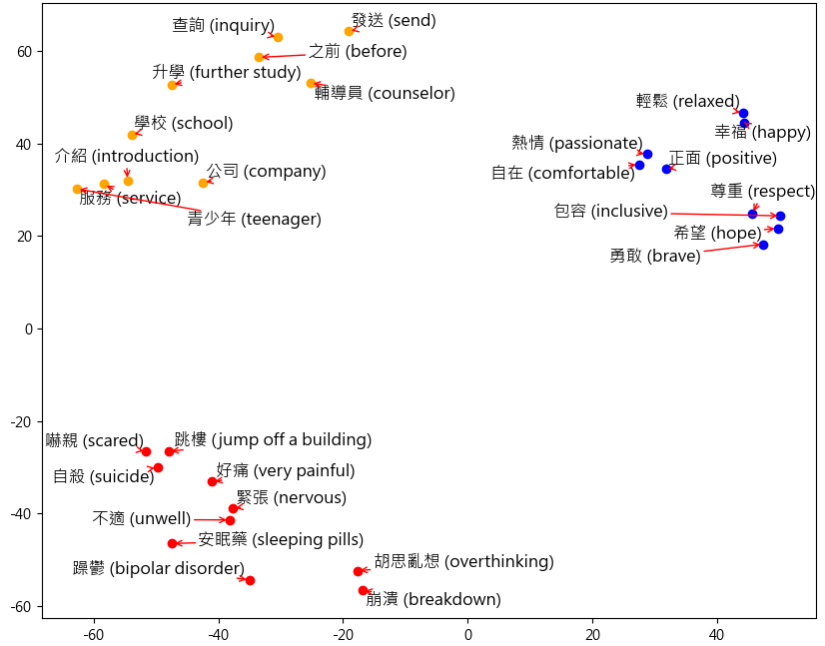
Distribution of Word2vec vectors among 30 sample words. To show the trained word2vec vectors, principal component analysis was used to reduce word2vec dimensions from 100-dimension to 2-dimension. The sampled 30 words were randomly selected to illustrate the performance of the trained Word2Vec model.

Second, to convert text to a machine-accessible form, word vector training is necessary. Word2Vec [25] is a popular model that can learn semantic knowledge from a large corpus without supervision and project words into a vector space in which the space distance among words represents their semantic relationships. Following previous studies using Open Up data to train the Word2vec model [7], the Python package *Gensim* (version 4.3.2; Python Software Foundation) was used for Word2vec processing. In particular, a Word2vec model with 5 as the window size and 100 as the vector size using Continuous Bag of Words was trained (Figure 4). The input was the 7,012,611 messages after word filtering.

Third, 80% of the data was randomly selected for training, and the remaining 20% was the test set.

Fourth, the baseline methods were selected for comparison with ChatGPT in terms of the following four criteria:

1. “Interpretability” refers to how easily one can understand and explain the outcome of a model. An interpretable model provides a transparent decision-making process, making it more trustworthy and easier to update.
2. “Efficiency” refers to the speed and computational resources required for training and deploying a model. An efficient model requires less time and computing power, making it easier to get the result with limited resources.
3. “Robustness” indicates the model’s resistance against overfitting, which means a model performs quite well on the training data but poorly on the new or test data. A robust model ensures consistent performance on new, unseen data, making it more reliable.
4. “Contextual memory over sequential data” emphasizes a model’s ability to process sequential data where the order and sequence are significant. A model that can retain contextual memory over sequential data means it reaches the final decision by considering the preceding and succeeding contexts of each entity and memorizing long-term dependencies over sequential data instead of treating input data as independent entities.

Table 2 contains description and advantages of the baseline methods.

The implementation details are provided in the subsequent paragraphs.

For LIWC, 2015 LIWC for the traditional Chinese (version 1.5; Pennebaker Conglomerates), which is a computerized text analysis method developed to examine the meaning of words, was used. It categorizes words into different linguistic and psychological dimensions based on a comprehensive dictionary.

For LR and SVM, the mean of word embeddings was used to generate message representations. The Python package *Scikit-learn* (version 1.3.0; Python Software Foundation) was imported to run logistic regression and SVM model, respectively. For SVM, a radial basis function kernel was used.

For LSTM, the mean of word embeddings was used to generate message representation. A detailed model structure can be found in the Multimedia Appendix 1. For the hyperparameter setting, the LSTM model was run multiple times with different learning rates, dropout rates, and batch sizes, and the optimal combination (ie, learning rate 0.005, dropout rate 0.5, and batch size 64) was selected, which caused the least training loss and overfitting problem. The model was trained using the categorical cross-entropy loss function with the Adam optimizer in 10 epochs, which were the best selections obtained through multiple training processes.

**Table 2:** Description and advantages of the 4 baseline methods.

Model	Description	Interpretability	Efficiency	Robustness	Contextual memory over sequential data
LIWC^a^	A text analysis program that counts words in psychologically meaningful categories	Categorizes words into psychological meaningful categories, making analysis interpretable and transparent	Allows for fast analysis of text and requires much less computational resource compared to other machine learning methods	Does not evolve over time, making it perform badly on new, unseen words	Treats each word as independent entity
LR^b^	A statistical method that predicts the likelihood of an outcome	Mathematically simple and inherently interpretable	More efficient compared to SVM^c^ and LSTM^d^	Less likely to overfit when appropriately regularized	Treats each word as independent entity
SVM	A type of algorithm that identifies patterns by drawing the best boundary through a “hyperplane” that separates different groups of data points	Less interpretable than LIWC and LR while more interpretable than LSTM	Less efficient than LIWC and LR while more efficient than LSTM	With proper class weights settings, SVMs can handle scenarios where data are imbalanced in different classes, which is robust to overfitting	Treats each word as independent entity
LSTM	A kind of recurrent neural network that can remember information for long periods through gated mechanism	Least interpretable compared to the other 3 models	Least efficient compared to the other three models	Designed specifically for vanishing gradient problem, ensuring its robustness against overfitting	Encompassed with gated mechanism, LSTM can retain memory from previous words in a sequence with variable length

^a^LIWC: Linguistic Inquiry and Word Count.

^b^LR: logistic regression.

^c^SVM: support vector machine.

^d^LSTM: long short-term memory.

## Results

### GPT-3.5 Versus GPT-4

We used 2 metrics, accuracy and *F*_1_-score, to evaluate the models’ performance (performance evaluation metrics in Multimedia Appendix 1). Accuracy represents the percentage of sentiment labels that the model correctly predicted out of all the sentiment labels it attempted to predict. However, this metric is biased toward sentiment labels with more data points (eg, if there are 90 neutral messages and 10 positive ones, a model that predicts all messages as neutral would have an accuracy of 90%). To measure performance in a way that is less sensitive to data imbalances, the *F*_1_-score, which represents the harmonic mean of the model’s precision and recall, was used to ensure that a model performs well across all 3 sentiment categories, not just the predominant one.

**Figure 5:**
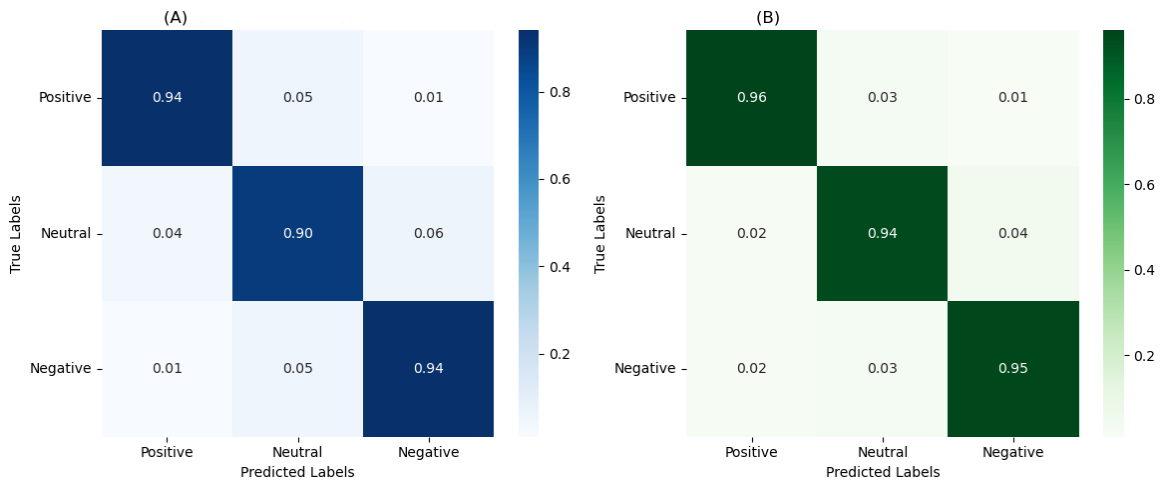
Confusion matrices for GPT-labeled sentiments (predicted labels) versus human-labeled sentiments (true labels). (A) The confusion matrix for GPT-3.5, and (B) the confusion matrix for GPT-4.

The results show that GPT-3.5 achieved 92.1% (5682/6169) accuracy and a 90.2% *F*_1_-score, and GPT-4 achieved 95.3% (5880/6169) accuracy and a 94% *F*_1_-score. Confusion metrics were used to better illustrate the results, in which rows represent the true sentiments annotated by human coders (true labels) and columns represent the sentiments predicted by the model (predicted labels). Figures 5A and 5B present the confusion metrics of GPT-3.5 and GPT-4. Darker colors reflect higher proportions of messages annotated by human coders with a particular label that received the same label from GPT, and vice versa for lighter colors. Figure 5A suggests that GPT-3.5 was more likely to classify “neutral” messages as “negative” (117/1943, 6% of neutral messages). In contrast, GPT-4 showed higher accuracy in detecting all 3 categories of sentiments. Both GPT-3.5 and GPT-4 outperformed other methods, and the McNemar test showed that GPT-3.5 and GPT-4 did not show perfect agreement (*P*=.01), which indicated that the results from GPT-4 were statistically significantly better than those from GPT-3.5.

### ChatGPT Versus Baseline Models

Table 3 reports the comparison between ChatGPT and the baseline models. LIWC had the lowest accuracy of 37.2% (2295/6169) and an *F*_1_-score of 24.2%, indicating its struggles in the sentiment classification task. Both the LR and SVM models showed accuracies of around 66%, but there was a noticeable difference in their *F*_1_-scores, with the LR model having a much higher score. This suggested that while SVM was almost as accurate as the LR model, it did not handle our imbalanced data as well as the latter. In addition, as LSTM can retain contextual memory over sequential data, it outperformed the traditional models with an accuracy of 70.9% (4374/6169) and an *F*_1_-score of 69.5%, indicating its better and more balanced performance at handling Cantonese sentiment classification among all the non-GPT baseline methods. Moreover, the results show that GPT-3.5, with a classification accuracy of 92.1% (5682/6169), and GPT-4, with an accuracy of 95.3% (5880/6169), outperformed the other state-of-the-art models for Cantonese sentiment classification tasks, with accuracies ranging from 37.2% (2295/6169) to 70.9% (4374/6169). Meanwhile, GPT-3.5 had an *F*_1_-score of 90.2% and GPT-4 reached an *F*_1_-score of 94%, indicating their balanced overall performance across the 3 sentiment categories, making them relatively more reliable.

**Table 3:** Comparison of ChatGPT and other state-of-art models in Cantonese sentiment analysis.

Model	Accuracy, n (%)	*F*_1_-score (%)
LIWC^a^	2295 (37.2)	24.2
LR^b^	4072 (66.0)	57.3
SVM^c^	4047 (65.6)	49.7
LSTM^d^	4374 (70.9)	69.5
GPT-3.5	5682 (92.1)	90.2
GPT-4	5880 (95.3)	94

^a^LIWC: Linguistic Inquiry and Word Count.

^b^LR: logistic regression.

^c^SVM: support vector machine.

^d^LSTM: long short-term memory.

## Discussion

### Overview

In this study, we tested whether the popular LLM ChatGPT could serve as a reliable sentiment classifier for Cantonese text. In a data set containing Cantonese text-based counseling session transcripts, we found that the 2 most recent variants of ChatGPT (GPT-3.5 and GPT-4) could accurately (>90%) detect sentiments expressed in Cantonese text. ChatGPT performed much better than lexicon-based methods and fine-tuned machine learning models at sentiment analysis. With a predefined list of words associated with specific categories, such as sentiments, lexicon-based text analysis methods are user-friendly and easy to interpret. However, these methods often achieve lower accuracy since they ignore the context in which words are used, which may lead to incorrect categorization of the text. Furthermore, the dictionaries are predefined and do not evolve over time, so they may lack the knowledge base needed for specific tasks. Compared with lexicon-based methods, ChatGPT is also user-friendly and much more accurate. With the rapid development of ChatGPT, in the future, there may be little need for lexicon-based methods such as LIWC. Moreover, ChatGPT is also a better choice than fine-tuned machine learning models. While machine learning classifiers require large amounts of manually annotated text to train and high coding proficiency, ChatGPT does not require extensive training data, and it is intuitive to use with little coding knowledge needed.

Cantonese sentiment analysis is particularly challenging for several reasons. First, Cantonese shares a writing system with Standard Chinese. The similarity can lead to misinterpretations of sentiments based on Standard Chinese sentiment analysis tools. Second, Cantonese has its own characters, expressions, and grammatical elements that may be overlooked by sentiment analysis models. Third, colloquialism and multilingualism in Cantonese pose great challenges to understanding sentiments in Cantonese text. Fourth, Cantonese has fewer NLP resources, such as labeled data sets or task-specific tools, making it difficult for robust NLP applications.

ChatGPT can tackle these challenges posed by Cantonese linguistic peculiarities. In our observations, ChatGPT can identify the sentiment of the same word with different meanings in Cantonese and Mandarin Chinese, which avoids the potential misinterpretation using Standard Chinese-specific models in the Cantonese context, which is a problem found in previous studies (eg, Xiang et al [20]). For example, “窩心,” meaning “feeling warm and caring” in Cantonese, was correctly classified as positive by ChatGPT, while other Standard Chinese-based machine learning models would label the word as negative due to its meaning “feeling uncomfortable and annoyed” in Mandarin Chinese. ChatGPT can also handle multilingual content accurately. For instance, the negative sentiment of “今日好down” (“feeling very down today”) with an English word “down” in a Cantonese sentence expressing feeling down or unhappy was accurately captured by ChatGPT. These mixed-language cases would be quite challenging for other traditional models. However, there might be some potential biases in using ChatGPT in Cantonese sentiment analysis. For example, GPT-3.5 at times interpreted the negative sentiment of being lost and not knowing how to address a problem as neutral. Moreover, GPT-3.5 tended to classify positive sentiments as neutral when a message expressed gratitude. We speculated that GPT-3.5 might have a linguistic bias in the Cantonese context; however, comparing the degree of bias against English was beyond the scope of this study, given that we had no comparable data in English for comparison. Moreover, ChatGPT has been shown to be biased in terms of gender and representation of minorities in the English context [26]. It is plausible that this bias might be exhibited in Cantonese as well.

The promising results of this study have 4 practical implications. First, it can assist in the quality control of services. By using ChatGPT for sentiment analysis in Cantonese, this study demonstrated the feasibility of using advanced LLMs to automatically classify sentiments in text-based counseling sessions. The accurate identification of sentiment can be used to assess the effectiveness of counseling interventions, improve good practice models, and enhance the overall quality of support offered to help-seekers. For example, as sentiment analysis has the potential to evaluate the counselor’s empathetic expressions, which were found to be predictive of positive counseling outcomes [9], ChatGPT could potentially help measure empathetic responses and affective synchronicity.

The second benefit is the accurate identification of emotional concerns and needs expressed by help-seekers during counseling sessions. By analyzing the sentiments conveyed in the text, counselors and service providers can gain deeper insights into the psychological well-being of their clients. This information can assist in detecting potential high-risk situations or individuals who may require timely attention or specialized care. For example, as the help-seeker’s suicide risk is closely correlated with their expressed sentiments [27], using ChatGPT to automatically analyze sentiments could potentially detect suicidal ideation at early stages and facilitate the counselor’s intervention accordingly.

In the long term, the implications of this study extend beyond the specific task of sentiment analysis in Cantonese text-based counseling services. The successful application of ChatGPT opens possibilities for using similar LLMs to tackle other tasks in the field of psychotherapy or NLP. These tasks may include more nuanced sentiment analysis, such as identifying discrete emotions to detect more specific expressed emotions (eg, happiness, anger, sadness, and fear). To achieve this goal, for example, an emotion-specific data set together with a codebook would be developed by Cantonese-speaking counselors and psychologists, where each piece of Cantonese text in the data set conveys one clear emotion to avoid ambiguity. The robustness and reliability of ChatGPT demonstrated in this study lay the foundation for further exploration and expansion of its capabilities in sentiment analysis and potentially other NLP-related tasks.

Finally, the success of Cantonese sentiment analysis hints at ground-breaking potential for tackling NLP tasks in other low-resource languages. This study’s approach and findings can be potentially applied to other low-resource language contexts due to the subsequent reasons. First, all low-resource languages face some common challenges, such as the lack of labeled data sets with domain knowledge and the lack of advanced NLP models. Second, ChatGPT was trained on various language resources, and studies such as Rathje et al [10] and Bang et al [11] suggested ChatGPT can, to a certain extent, understand low-resource languages. Thus, with adjustments to account for linguistic and cultural differences, our methodology is transferable and scalable to other low-resource languages. More generally, this study could help shape current approaches to NLP tasks in low-resource languages. This innovation might bring the cutting-edge benefits of NLP technology closer to billions of people worldwide who speak these languages, thus bringing about immense immediate benefits.

However, since counseling sessions are inherently sensitive and private, using LLMs like ChatGPT to analyze sentiment in a counseling context raises several ethical considerations. First, data privacy is of major concern. The use of LLMs to analyze counseling session data must ensure anonymity and confidentiality and accord with ethics approval. Second, the decision-making process of LLMs is not fully interpretable, especially since we do not know what materials were provided during the training stage, which poses challenges in justifying their outputs. Third, LLMs might generate biased statements or unsafe advice, which should receive more attention, particularly in sensitive settings like counseling. Addressing these ethical considerations enables us to advance our research responsibly.

### Limitations and Future Directions

While our findings are promising, this study has a number of limitations. First, although our annotation codebook was comprehensive, the labeled data was imbalanced. With only 959 out of 6169 cases (15.5%) belonging to “positive” sentiment, we may lack a comprehensive understanding of the positive affect expressed in counseling sessions. In the future, sessions with more messages expressing positive sentiment will be included to ensure data balance. Second, the generalizability of our findings is limited by the data source. The way of expressing sentiment in different domains, for example, social media and customer feedback, could differ. As this study focused on counseling text, future research could explore various text sources to enhance the generalizability of this study. Third, we observed that there were still some biases and limitations to using ChatGPT in Cantonese sentiment analysis.

Future work can consider several directions. First, researchers can consider using different prompting strategies, such as few-shot learning (ie, providing several labeled examples in the prompts), to further enhance the performance of ChatGPT in Cantonese sentiment analysis tasks. Few-shot learning provides additional references and materials on top of the training data set for GPT to know the context and make better predictions. The second possible direction is to use ChatGPT in more types of sentiment analysis tasks, such as discrete emotion detection, to detect more specific expressed emotions (eg, happiness, anger, sadness, and fear). The third direction future work can consider is to go beyond sentiment analysis tasks and to use ChatGPT in other NLP-related tasks, such as text summarization tasks (ie, to generate concise summaries of long texts such as counseling session transcripts) and translation tasks (ie, translating languages while taking into consideration linguistic and cultural differences).

### Conclusions

We evaluated ChatGPT’s performance in analyzing sentiments in Cantonese web-based counseling transcripts and compared its accuracy against other mainstream methods. Our results suggest that not only is ChatGPT a reliable tool for detecting sentiments in Cantonese text, but it has also surpassed the accuracy of existing sentiment analysis methods, such as lexicon-based methods and fine-tuned machine learning models, with many additional advantages, such as the use of straightforward and intuitive prompts. The findings could have many practical implications, including improving the quality of counseling services, providing timely support for help-seekers, using LLMs to tackle other psychology or NLP-related tasks, and bringing cutting-edge NLP tools to low-resource languages.

## Appendix

Multimedia Appendix 1 Supplementary materials.

## Abbreviations

AI: artificial intelligence
LIWC: Linguistic Inquiry and Word Count
LLM: large language model
LR: logistic regression
LSTM: long short-term memory
LSTM-SAT: long short-term memory sentiment augmented attention network
NLP: natural language processing
PLM: pretrained language model
SVM: support vector machine

## Data Availability

Given its sensitivity, the raw transcript data are not publicly available but are available from the corresponding author on reasonable request. The code used to perform the analysis can be found on GitHub [28].
