# Supplementary material for: Efficacy of ChatGPT in Cantonese Sentiment Analysis: Comparative Study
Source: J Med Internet Res. 2024 Jan 30;26:e51069. doi: 10.2196/51069 (PMC10865189; doi:10.2196/51069)
Supplement: Multimedia Appendix 1 [file jmir_v26i1e51069_app1.docx]

**Supplementary Materials**

**Efficacy of ChatGPT in Cantonese Sentiment Analysis: Comparative Study**

Table of Contents

[Codebook 1](#_Toc151977525)

[Baseline methods implementation 4](#_Toc151977526)

[Performance evaluation metrics 8](#_Toc151977527)

#

# Codebook

Table 1. Codebook for human annotation

| **Sentiment** | **Definition** | **Tips** | **Examples** |
| --- | --- | --- | --- |
| **Positive** | Positive sentences describe feelings of pleasure, gratitude, satisfaction, compliment, or recommendation. Emojis and punctuation can be indicative of positivity. | Affirm the help-seeker’s strengths and motivations. Provide reassurance and encouragement. | 你咁樣已經做得好好 (You have done very well) |
|  |  | Utterances that involve setting goals, making plans or express intentions to change or improve the situation | 我都好想改變 (I really want to change) |
| **Neutral** | The neutral label is used for any sentences the annotator could not identify as positive or negative. Factual sentences that do not present sentiment orientation, such as “I am 25 years old” and sentences the annotator cannot completely comprehend were annotated as neutral. Sentences that are routine replies from the counselor, such as ‘Is there anything you want to share with me today?’ were also labeled as neutral. | Describe experiences that contribute to the appealing problem | 我同男朋友拍左八年拖 (I have been dating with my boyfriend for 8 years) |
|  |  | Ask for information, seek help, advice, affirmation, or reassurance | 不如你講多少少你同屋企人嘅相處我聽呀 (Why don’t you tell me more about the way you get along with your family?) |
|  |  | Concise rephrasing of the other person’s statements | 你突然發現一個咁親密既人背叛自己 仲要自己去挽留對方 (You suddenly found that someone so close had betrayed you and you had to try to salvage the relationship) |
|  |  | Reflection of feelings | 明白你呢刻好傷心 (I understand you feel very sad right now) |
|  |  | Provide suggestions and plans about how to change | 不如都比多啲時間空間自己呢 先讓自己好好休息啊 (It is better to give yourself more time and space. Take a good rest first.) |
|  |  | Provide useful information, such as factual data, referral services | 你可以試下聯絡呢度 呢度都有幾個關於戒賭既專業機構 (You can try to contact here. There are several professional organizations related to quitting gambling) |
|  |  | Greetings in the beginning and saying goodbyes in the end | 如果您日後有需要，歡迎您再入嚟平台搵我哋再傾 (If you have any needs in the future, you are welcome to come back to the platform and find us for further discussions) |
| **Negative** | Negative sentences describe feelings of disagreement, sadness, complaint, helplessness or hate. Factual information such as the description of the help-seeker’s sad issues is also considered negative. Emojis and punctuations are also indicative of negativity. | Nervousness | 我而家好緊張 (Now I am very nervous) |
|  |  | Hopelessness | 好似都冇可能做到 (It seems that it cannot be done) |
|  |  | Restlessness or fidgety | 我都俾佢搞到好煩躁 (He made me so annoyed) |
|  |  | Depression | 我成日好抑鬱沮喪 (I am depressed and frustrated all day) |
|  |  | Worry | 我好擔心你 (I am so worried about you) |
|  |  | Worthlessness | 全世界都唔鍾意我 (Nobody likes me) |

The rules to annotate a message with multiple sentences: First, a message is divided into separate sentences. Second, each sentence’s sentiment is annotated. Third, the overall sentiment of a message is determined by a predefined rule listed in table below.

Table 2. Rules to label a message with multiple sentences.

| **Sentiments of multiple sentences within one message** | **Overall Sentiment of the message** |
| --- | --- |
| Positive + Neural | Positive |
| Negative + Neutral | Negative |
| Positive + Negative | If the number of positive sentences is equal to the number of negative sentences, assign weights to sentences based on their position and emphasis. For example, the sentiment of the concluding sentence determines the overall sentiment of the message.  If the number of positive sentences is not equal to the number of negative sentences, the majority sentiments determine the overall sentiment. |
| Positive + Negative + Neutral | Sentences with neutral sentiment are removed first. Then, the rule is the same as the “Positive + Negative” scenario. |

# Baseline methods implementation


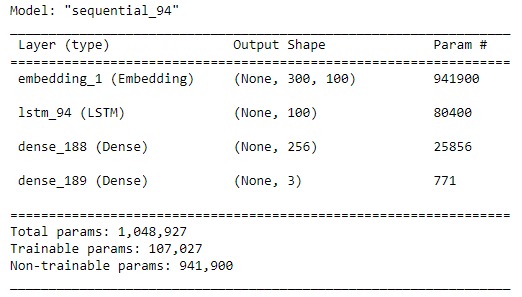


Figure4: LSTM model structure


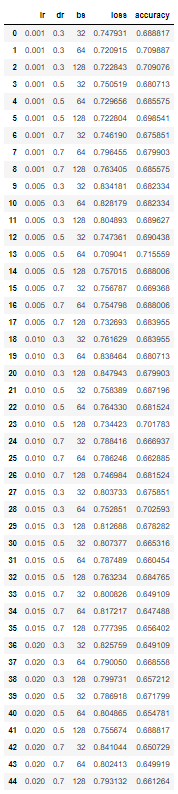


Figure5: LSTM training process using different combinations of learning rate, dropout rate and batch size


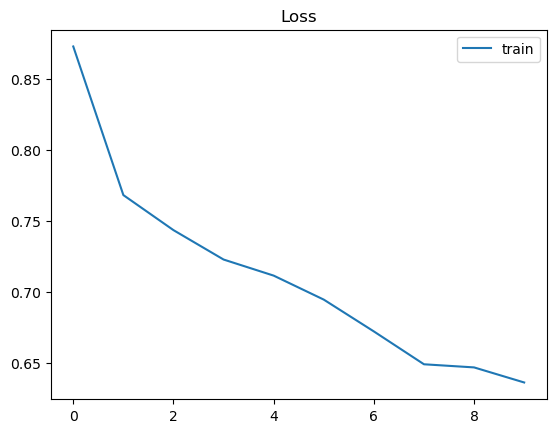


Figure6: LSTM loss during training process with the best hyperparameter selections (i.e., learning rate = 0.005, dropout rate = 0.5, batch size = 64)


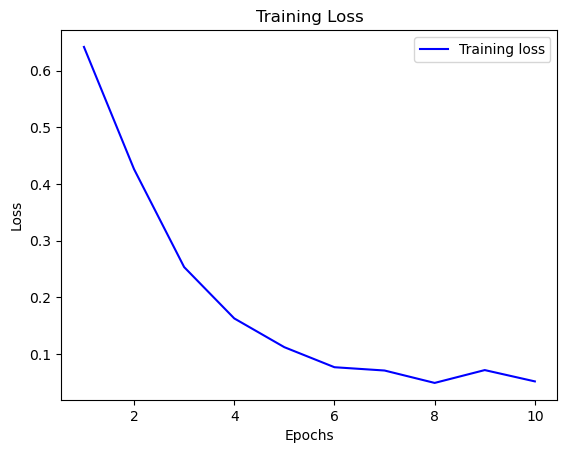


Figure7: BERT loss during training process

# Performance evaluation metrics

$True Positives \left( TP \right).$The number of items correctly labeled as having the positive condition (e.g., correctly identified as positive sentiment).

$True Negatives \left( TN \right).$ The number of items correctly labeled as not having the positive condition (e.g., correctly identified as neutral or negative sentiment when they are not positive).

$False Positives \left( FP \right).$ The number of items wrongly labeled as having the positive condition (e.g., incorrectly identified as positive sentiment when they are neutral or negative).

$False Negatives \left( FN \right).$ The number of items wrongly labeled as not having the positive condition (e.g., incorrectly identified as neutral or negative sentiment when they are positive).

$Accuracy$. The metrics of accuracy represents the percentage of sentiment labels that the model correctly predicted out of all sentiment labels the model attempted to predict.

$$Accuracy=\frac{TP+TN}{TP+FP+TN+FN}$$

$F1 score$. Macro-averaged F1 score was used to quantify classification accuracy in a way that is less sensitive to data imbalances.

$$F1=2\times\frac{Precision\times Recall}{Precision+Recall}$$

$Precision$. Precision represents the proportion of data points labeled with the given class by the model that are truly of that class (True Positives) against falsely labeled (False Positives).

$$Precision= \frac{TP}{TP+FP}$$

$Recall$. Recall represents the percentage of True Positives over the True Positives and False Negatives.

$$Recall=\frac{TP}{TP+FN}$$
